# PD170 Modification Of The All Wales Medicines Strategy Group Appraisal Process Provides Faster Access To Children’s Medicines In Wales

**DOI:** 10.1017/S0266462324003969

**Published:** 2025-01-07

**Authors:** Kelly Wood, Alice Varnava, Ruth Lang, Anthony Williams, Professor James Coulson, Andrew Champion, Claire Ganderton

## Abstract

**Introduction:**

Medicines routinely funded for use in Wales undergo health technology appraisal by the All Wales Medicines Strategy Group (AWMSG) or the National Institute for Health and Care Excellence (NICE). This includes pediatric license extensions (PLE) notwithstanding any existing advice in adults. A review of the PLE process was conducted with the aim of providing faster access to children’s medicines in Wales.

**Methods:**

Data were collected for PLE appraisals of medicines previously approved for adults by the AWMSG or NICE that subsequently went through the original PLE process between January 2010 and December 2020, or a simplified PLE process between January 2021 and March 2023. Data were analyzed using descriptive statistics and a two-tailed t-test (unequal variance) to test the null hypothesis that the difference between the two means was zero. An alpha of less than 0.05 was considered significant. Feedback was obtained from relevant stakeholders including the Association of the British Pharmaceutical Industry (Wales) and the Royal College of Paediatrics and Child Health.

**Results:**

The AWMSG issued positive recommendations for all PLE appraisals included in the data collected, and these were endorsed by the Welsh Government. Appraisals that went through the original PLE process (n=56) took a mean 229.8 days (standard deviation 55.6), whereas those that went through the simplified PLE process (n=15) took a mean 102.6 days (standard deviation 48.1; p < 0.0001). The rapid access to children’s medicines was welcomed by the Association of the British Pharmaceutical Industry and the Royal College of Paediatrics and Child Health.

**Conclusions:**

Review of the 2020 and 2023 PLE processes facilitated faster access to clinically effective and cost-effective medicines for children in Wales. In March 2023, the AWMSG and the Welsh Government reviewed these results and agreed that because all PLE medicines were approved for use within Wales irrespective of the process used, the AWMSG would no longer be required to routinely appraise PLEs.

